# The stem cell-associated Hiwi gene in human adenocarcinoma of the pancreas: expression and risk of tumour-related death

**DOI:** 10.1038/sj.bjc.6604653

**Published:** 2008-09-09

**Authors:** L F Grochola, T Greither, H Taubert, P Möller, U Knippschild, A Udelnow, D Henne-Bruns, P Würl

**Affiliations:** 1 Department of General, Visceral and Transplantation Surgery, The University of Ulm, Steinhövelstrasse 9, Ulm 89075, Germany; 2 Institute of Pathology, Martin-Luther-University Halle-Wittenberg, Magdeburger Str. 14, Halle 06097, Germany; 3 Institute of Pathology, The University of Ulm, Albert-Einstein-Allee 11, Ulm 89081, Germany

**Keywords:** Hiwi, prognosis, pancreatic carcinoma, tissue microdissection

## Abstract

Piwi proteins and their interaction with piRNAs have rapidly emerged as important contributors to gene regulation, indicating their crucial function in germline and stem cell development. However, data on the Hiwi 1 (Hiwi) gene, one of the four human Piwi homologues, are still scarce. Therefore, we investigated the Hiwi mRNA expression in microdissected PDAC tissues from patients with ductal adenocarcinoma of the pancreas (PDAC) by quantitative real-time PCR and the protein expression by immunohistochemistry. Elevated levels of Hiwi mRNA transcripts were measured in 40 out of 56 tissues and a positive immunostaining of Hiwi was detected in tumours of 21 out of 78 patients. There was no general impact of elevated Hiwi mRNA transcript levels or protein expression on survival, as tested by multivariate Cox regression and Kaplan–Meier analysis. However, men showed a significantly increased risk for tumour-related death in case of down- or upregulated expression of Hiwi mRNA (relative risk (RR)=2.78; *P*=0.034). In summary, we report the first analysis of Hiwi expression in PDAC and its impact on prognosis. We suggest that alterations in mRNA expression of Hiwi can increase the risk of tumour-related death in male PDAC patients.

Current stem cell genetic research provides only scarce data on Hiwi 1 (Hiwi) – one of four human homologues of the Piwi gene family – despite growing focus of studies on the latter and its interacting RNAs (piRNAs), for which a key function in transcriptional gene silencing and germline/stem cell development in flies and mammals has been demonstrated (Aravin *et al*, 2007; Seto *et al*, 2007; Farazi *et al*, 2008). Data on Piwi proteins, a subfamily of Argonaute proteins, highlight their impressive biological capacities, such as biogenesis/regulation of small RNAs, control of protein synthesis and mRNA stability, showing conserved functions in maintenance and self-renewal of stem cells (Seto *et al*, 2007; Hutvagner *et al*, 2008).

It has been suggested that deregulation of stem cell self-renewal may cause the development of malignancies because normal stem cells share several similarities with so-called tumour stem cells, for example, the ability to self-renew and a relative resistance to drugs (Soltysova *et al*, 2005; Chen *et al*, 2007; Taubert *et al*, 2007a). Besides being expressed abundantly in germline cells of human testes, Hiwi's enhanced expression was detected in testicular seminomas, which originate from embryonic germ cells with retention of a germ cell phenotype (Qiao *et al*, 2002). This has given rise to the assumption that Hiwi overexpression may be involved in the development of germ cell malignancy, prompting further investigation on other human malignancies. Consequently, it has been shown that expression of the Hiwi gene in human gastric cancer was associated with the proliferation of cancer cells (Liu *et al*, 2006). Moreover, in patients with soft-tissue sarcoma, elevated Hiwi mRNA transcript levels were associated with a significantly increased risk of tumour-related death (Taubert *et al*, 2007a, 2007b). However, Hiwi was not detectable in leukaemia cell lines (Sharma *et al*, 2001).

Ductal adenocarcinoma of the pancreas (PDAC), a dismal disease with a late clinical presentation and a very poor overall prognosis (Yeo *et al*, 2002), seems to be an interesting subject to investigate with regards to the involvement of stem cell-associated genes and Hiwi in particular. The main features of PDAC–aggressive biological phenotype, early local invasion with high metastatic potential and a high resistance to radiation and chemotherapy (Yeo *et al*, 2002)–suggest the involvement of cells with stem cell characteristics in PDAC. Analysis of the stem cell-associated gene Hiwi in this malignancy might provide important clues to the understanding of this disease.

Besides the above listed tumour entities, no studies on other malignancies, particularly on PDAC, on the involvement of Hiwi expression in tumour development have been reported, and the impact of Hiwi expression on patients' prognosis has only been studied in soft-tissue sarcomas yet (Taubert *et al*, 2007a, 2007b). Therefore, the aim of our study was to investigate the expression profile of Hiwi and its impact on prognosis in PDAC patients.

## Materials and methods

### Patients

We analysed a cohort of patients who underwent primary surgery for PDAC in the years 2001–2005 in our clinic (Department of Surgery 1, University of Ulm, Germany). Paraffin-embedded tissue of 78 patients (31 females and 47 males, age range 34–80 years; mean age 61.1 years) was obtained. Furthermore, fresh-frozen tissue of 56 patients (22 females and 34 males, age range 34–80 years; mean age 61.7 years) was conserved. All patients from whom fresh-frozen tissue suitable for microdissection (sufficient amount of neoplastic cells per sample (total of >4000 cells per patient) and intact RNA (as tested with Agilent Bioanalyzer, see below) was obtained were included in the study. The mean observation time was 15.99 (range 1–61) months and the median survival rate was 15.26 (range 1–52) months. Four patients died from non-tumour-related causes. None of the patients has received neoadjuvant chemo- or radiotherapeutic treatment. All patients included in the study gave written informed consent. The approval of the local ethics committee was obtained.

### Microdissection and QRT–PCR analysis

Fresh-frozen tissue samples from 56 PDAC patients were microdissected to exclude stromal tissue and highly enrich neoplastic cells (Burgemeister *et al*, 2003). Surgical pancreatic resection specimens were immediately placed on ice and subsequently snap-frozen, then stored at −80°C. The integrity of the isolated RNA (using Innuprep RNA mini kit, AJ Innuscreen GmbH, Berlin, Germany) was confirmed with the Agilent 2100 Bioanalyzer (Agilent Technologies, Santa Clara, CA, USA). The cryostat tissues were cut into 8–10-*μ*m sections. Microdissection of selected areas (containing approximately 50–300 neoplastic ductal epithelial cells per dissected area, >4000 cells per patient per tissue) was carried out by Laser microdissection and pressure catapulting technique (PALM Microlaser Technologies, Bernried, Germany) on cresyl violet-stained sections (Supplementary Figures). Subsequently, we performed quantitative real-time PCR analysis for the expression of Hiwi mRNA. The mRNA expression of Hiwi was quantified by a commercially available TaqMan gene expression assay (Assay-ID: Hs01041737_ml; Applied Biosystems, Foster City, CA, USA) and standardised to the transcript level of hypoxanthin-phospho-ribosyl-transferase (HPRT), using TaqMan gene expression assay (Assay-ID: Hs99999909_ml; Applied Biosystems).

### Immunohistochemistry

Analysis of 78 PDAC tissue specimens by immunohistochemistry (IHC) was performed. All specimens were fixed in buffered formalin and embedded in paraffin. Sections of 4-*μ*m thickness were deparaffinised in xylene and rehydrated. After microwave-heating of the sections in citrate buffer at 450 W for 2 min and subsequently at 80 W for 20 min, slides were cooled to room temperature, then treated with peroxidase-blocking reagent (DakoCytomation, Dako, Hamburg, Germany) and serum-blocking reagent (VECTASTAIN Elite ABC Kit; Vector Lab., Burlingame, CA, USA). Sections were incubated overnight at 4°C with an 1 : 250 diluted goat anti-human polyclonal Hiwi antibody (HIWI N-17:sc-22685; Santa Cruz Biotechnology Inc., Santa Cruz, CA, USA). The slides were further treated with the VECTASTAIN Elite ABC Kit, according to the manufacturer's instructions using a secondary biotinylated Anti-goat-IgG antibody. Diaminobenzidine was used as chromogen and the sections were counterstained with Mayer haematoxylin. All washing steps were performed in Tris-buffer. Immunohistochemical evaluation was carried out by at least two independent investigators. At least 500 neoplastic ductal cells were counted/examined per tissue section. Positive staining required a minimum of 10% of neoplastic cells exhibiting a distinct staining. Three patterns of staining intensity were defined: strong, intermediate and weak staining. Testicular seminomas served as positive control.

### Statistical analysis

Kaplan–Meier and multivariate survival analysis according to the Cox's proportional hazards regression model (adjusted for tumour staging, type of tumour resection, patients' age and gender) was performed for evaluation of Hiwi mRNA expression and IHC results. First, two categories of Hiwi levels were set up for analysis of mRNA transcript levels: (1) ‘No expression’ (<0.001 attogram (ag) Hiwi mRNA per (femtogram) fg HPRT mRNA); (2) ‘Elevated expression’ (⩾0.001 ag Hiwi mRNA per fg HPRT mRNA). Second, analyses using the mean and median values as cutoff levels were performed. Accordingly, threshold levels were set at 1.12 and 0.62 ag Hiwi mRNA per fg HPRT mRNA, respectively. A third analysis applied the quartile of Hiwi mRNA expression and resulted in four groups, that is, two groups with low expression (<0.061 ag Hiwi mRNA per fg HPRT mRNA; *n*=27), a group with intermediate expression (intermediate expression ⩾0.061–<0.568 ag Hiwi mRNA per fg HPRT mRNA; *n*=15) and a group with high expression (⩾0.568 ag Hiwi mRNA per fg HPRT mRNA; *n*=14). In this analysis, we combined groups with the low and high expression profile and compared their joint expression with the intermediate expression group. The SPSS 15.0 software was used for statistical analysis. Values of *P*<0.05 were considered significant.

## Results

### Pattern of Hiwi expression

Elevated transcript levels of Hiwi mRNA were measured in 40 out of 56 PDAC tissues (71.4%), whereas the remaining patients showed no detectable transcripts. The mean expression level of Hiwi transcripts was 1.12 (range: 0.00–28.24; median: 0.62) ag Hiwi mRNA per fg HPRT mRNA. Patients whose tumours showed an expression below or above the mean or median, revealed an equal distribution between tumour stages, resection types (R-status), age and gender (Table 1).

**Figure 1:**
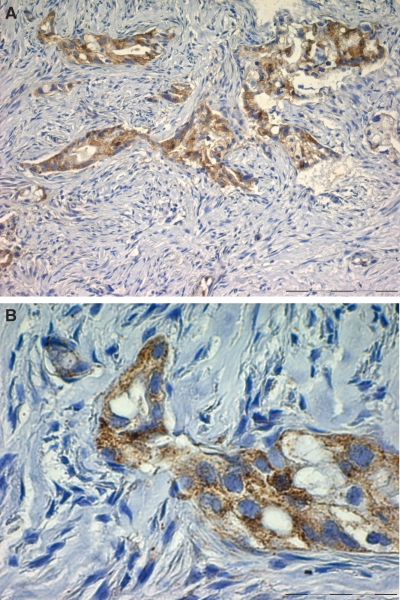
Immunohistochemical staining for Hiwi in ductal adenocarcinoma of the pancreas. Cytoplasmatic expression in neoplastic ductal carcinoma cells. (**A**: × 100; **B**: × 400).

In IHC, positive immunostaining with the anti-HIWI N-17 antibody was detected in 21 out of 78 samples (26.9%), whereas the remaining 57 tumours showed no positive staining in more than 10% of the cells (Table 2). Of the 21 positively stained tumours, 11 (14.1%) showed a simultaneous immunostaining in the nucleus and cytoplasm of the neoplastic ductal cells and 10 (12.8%) demonstrated a sole cytoplasmatic staining pattern (Figure 1). A total of 17 tumours showed a strong and 4 a moderate staining intensity, whereas a weak staining was not observed among the tumour samples. Patients with a Hiwi expression on protein level showed an equal distribution between the tumour stages, resection types, age and gender.

### Clinical outcome and Hiwi expression

**Figure 2:**
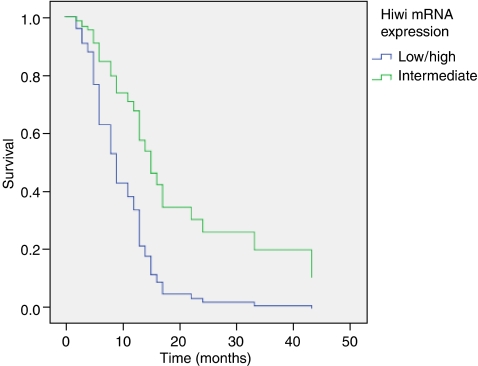
Multivariate Cox's regression analysis: Hiwi mRNA expression in two male patient groups (adjusted to tumour stage and type of resection) in ductal adenocarcinoma of the pancreas: a group that includes patients whose tumours showed low or high expression of Hiwi mRNA, (*n*=24) carries a 2.78-fold increased risk of tumour-related death (*P*=0.034) compared to patients whose tumours had an intermediate Hiwi transcript level (*n*=10).

In Kaplan–Meier and multivariate Cox's regression hazard analysis (adjusted to tumour resection type and tumour stage) no impact of Hiwi mRNA expression on survival was determined in a comparison of groups of patients whose tumours expressed Hiwi either below or above the median/mean level of expression. Furthermore, separation of patients in two groups with tumours expressing undetectable levels (<0.001 ag Hiwi mRNA per fg HPRT mRNA) or detectable levels of Hiwi transcripts (⩾0.001 ag Hiwi mRNA per fg HPRT mRNA) did not show an impact of Hiwi expression on survival (RR=1.401; *P*=0.328). These findings were independent of sex or age. In a former study involving patients with soft-tissue sarcoma, we identified three groups of patients, where patients with an elevated or a reduced Hiwi expression in their tumours showed a poor prognosis compared to patients with an intermediate expression level (Taubert *et al*, 2007a). Therefore, we separated patients into a group where tumours carried a high or a low expression of Hiwi transcripts and a group with intermediate Hiwi expression. We performed this separately for female and for male patients. In a multivariate Cox's regression analysis in male patients, we detected a 2.78-fold increased risk of tumour-related death for patients (*P*=0.034) whose tumours showed an elevated or a decreased expression of Hiwi compared to patients with an intermediate expression of Hiwi (Figure 2). Interestingly, for female patients no different impact on prognosis could be found comparing both patient groups.

Evaluating the results obtained from immunohistochemistry, no impact of Hiwi protein expression on tumour-related death was found. No effect on patients' prognosis was observed either for isolated cytoplasmatic staining or simultaneous staining of the nucleus and cytoplasm. There was no significant impact on the patients' gender and Hiwi protein expression on survival, as evaluated by IHC.

## Discussion

Here, we report the first analysis of Hiwi mRNA and protein expression in PDAC and its relation to prognosis. Consistent with findings on other tumour entities, our results indicate that Hiwi might not only be relevant in germ cell malignancies but also in solid tumours of epithelial or mesenchymal origin (Qiao *et al*, 2002; Liu *et al*, 2006; Taubert *et al*, 2007a, 2007b). Besides testicular tumours, so far the expression of the human homologue of the Piwi gene has only been investigated in leukaemia cell lines (Sharma *et al*, 2001), gastric cancer (Liu *et al*, 2006) and soft-tissue sarcomas (Taubert *et al*, 2007a, 2007b), demonstrating elevated protein and/or mRNA transcript levels in the two latter kinds of tumours. Elevated and reduced Hiwi mRNA message were significantly associated with a poor outcome in soft-tissue sarcoma patients, outlining its possible key function in non-germ cell malignancies (Taubert *et al*, 2007a, 2007b). Accordingly, we observed a high detection rate of Hiwi mRNA transcripts in our cohort, with elevated mRNA levels detectable in 40 out of 56 patients.

Regarding survival analysis, no impact of Hiwi mRNA expression on tumour-related death was ascertained for all PDAC patients. However, when only male patients were considered, a 2.78-fold increased risk of tumour-related death was detected for patients whose tumour showed an altered, that is, down- or upregulated expression of Hiwi mRNA compared to those with an intermediated Hiwi transcript level. The gender-dependent impact of Hiwi mRNA expression on prognosis is not easy to explain. Hiwi expression has been detected in normal human testes and male germline malignancies, but was not detectable in ovaries (Qiao *et al*, 2002). Moreover, mice bearing targeted mutations in the murine-homologues Miwi (Deng and Lin, 2002), Mili (Kuramochi-Miyagawa *et al*, 2004) and Miwi2 (Girard *et al*, 2006) are male-sterile with distinct defects in spermatogenesis. However, in lower eukaryotic organisms, such as *Drosophila*, mutations of the Piwi gene caused failure in germline stem cells in both females and males (Cox *et al*, 1998). Hormonal aspects might have a function in PDAC as investigations on the expression of androgen and oestrogen/progesterone receptors have demonstrated (Andren-Sandberg *et al*, 1999; Konduri *et al*, 2007; Konduri and Schwarz 2007; Lam *et al*, 2008). It would be interesting to investigate the expression of the Hiwi gene – especially in tumours of hormonally regulated or active organs – as it might give additional clues to its function and regulative processes in humans.

As far as immunohistochemistry for Hiwi protein expression is concerned, a strong positive staining in 26.9% of the carcinomas (21 out of 78 patients) was observed, a rate substantially lower than for mRNA transcript levels. These differences in the rates of elevated Hiwi expression between mRNA and protein levels, however, are very likely to be because of the much higher sensitivity of the real-time PCR technique than that of IHC, thus not allowing for detection of relevant Hiwi protein levels. Furthermore, a wide range of modified protein variants (e.g. post-translational or other downstream modifications) might remain undetected by IHC.

The detection rate of Hiwi mRNA transcripts and the association with a gender-dependent risk pattern underscores the involvement of stem cell-associated genes in carcinogenesis and cancer development in PDAC. Given the tumour's aggressive potential and frequent changes in the Hiwi mRNA levels, Hiwi as a stem cell-associated gene may have general and early impact on tumourigenesis in PDAC. A comparison of expression levels in PDAC and in the normal pancreas could help to relate expression levels to tumour biological behaviour. Altogether, the gene might be a potential important target for further investigation in carcinogenesis and therapy of this dismal disease.

In summary, we demonstrate for the first time the expression of the stem cell-associated Hiwi gene – a human homologue of the Piwi family – in human PDAC and identify Hiwi as a potential target for further investigation in carcinogenesis and therapy of this dismal disease. The detection rate of Hiwi mRNA in clinically apparent advanced PDAC and the lack of the gene's significant impact on tumour-related death, in general, might be indicative of the tumour's aggressive potential and hints at a broad and early impact of the Hiwi gene in carcinogenesis of PDAC. Intriguingly, alterations in mRNA expression levels of Hiwi can increase the risk of tumour-related death in male patients, pointing at gender differences in the prognostic impact of Hiwi mRNA expression in PDAC.

## Figures and Tables

**Table 1:** Hiwi mRNA: clinical and histopathological data of ductal adenocarcinoma of the pancreas patients

				**Stage**					
**Patient no.**	**Age at surgery**	**Sex**	**Survival (months)**	**T**	**N**	**M**	**R-status**	**Grading**	**Hiwi mRNA (in ag/fg HPRT)**
1	59	M	13	3	1	0	0	2	28.241
2	53	F	16	3	1	0	0	2	0.038
3	60	F	21	3	0	0	0	2	1.366
4	64	M	Alive^a^	3	0	0	1	3	0.139
5	40	F	19	3	1	0	0	3	0.010
6	59	M	24	3	1	0	0	3	0.979
7	47	F	20	3	1	0	0	2	0.072
8	46	F	15	3	1	0	0	2	1.825
9	71	M	5	3	0	0	1	3	0.576
10	66	F	4	4	1	0	2	1	0.000
11	70	M	15	3	1	0	1	3	7.689
12	67	M	6	3	1	0	0	2	0.000
13	74	M	3	4	1	1	2	1	0.523
14	75	F	49	3	1	0	0	2	1.139
15	49	M	22	3	1	0	0	1	1.112
16	70	M	4	3	1	0	0	3	2.526
17	68	M	11	3	0	0	0	3	0.000
18	72	F	33	3	1	0	1	2	0.935
19	73	F	1	3	1	0	0	1	0.000
20	58	M	43	3	2	0	0	2	0.077
21	74	M	Alive^a^	3	1	0	0	3	0.003
22	69	F	15	4	2	1	2	3	0.060
23	43	M	2	3	2	1	2	3	0.000
24	56	M	5	4	2	1	2	2	0.069
25	48	M	9	2	1	0	0	3	0.000
26	70	M	8	4	2	1	2	2	0.141
27	76	M	15	3	0	0	0	2	0.063
28	57	F	3	4	0	0	0	3	0.053
29	61	M	14	2	1	0	0	3	0.030
30	69	M	9	3	1	0	0	2	0.545
31	47	M	2	3	1	0	0	3	5.160
32	52	F	29	3	0	0	0	2	0.000
33	38	F	4	4	2	1	2	1	0.112
34	65	M	16	3	0	0	0	3	0.074
35	75	M	13	3	0	0	0	2	0.010
36	60	F	4	3	1	1	1	3	0.015
37	61	F	37	3	1	0	0	2	0.005
38	62	F	16	3	0	0	1	2	0.000
39	80	M	33	3	1	0	0	1	0.000
40	69	F	1	3	1	0	0	2	0.027
41	42	M	3	4	2	1	2	3	0.050
42	52	M	Alive^a^	3	1	0	0	3	0.000
43	52	M	13	3	1	0	0	3	0.000
44	71	F	23	3	0	0	0	2	2.374
45	64	F	Alive^a^	1	1	0	0	2	0.000
46	69	M	Alive^a^	3	1	0	0	1	0.109
47	68	F	Alive^a^	2	0	0	0	2	0.203
48	34	M	6	3	1	0	1	3	0.000
49	61	F	12	2	0	0	0	3	0.114
50	71	M	12	3	0	0	0	2	0.000
51	66	M	17	3	1	0	0	2	2.920
52	71	F	11	3	1	0	2	3	3.213
53	67	M	17	3	1	0	0	3	0.182
54	67	M	6	3	1	0	1	3	0.000
55	69	M	5	3	1	1	2	2	0.000
56	55	M	8	3	1	0	1	3	0.016

a At follow-up.

**Table 2:** Immunohistochemistry – summary of clinical and histopathological data

	**Total (*n*=78)**	**HIWI staining^a^**			**Patients at follow-up**	
		**CP (*n*=10)**	**CP/nucleus (*n*=11)**	**No staining (*n*=57)**	**Alive^b^** **(*n*=10)**	**Dead^c^** **(*n*=68)**
**Men/women**	**47/31**	**7/3**	**6/5**	**34/23**	**5/5**	**42/26**
*Tumour stage (%)*						
I	6	1 (16.7%)	2 (33.3%)	3 (50.0%)	2 (33.3%)	4 (66.6%)
II	12	1 (8.3%)	0 (0%)	11 (91.6%)	2 (16.7%)	10 (83.3%)
III	53	7 (13.2%)	7 (13.2%)	39 (73.6%)	6 (11.3%)	47 (88.7%)
IV	7	1 (14.3%)	2 (28.6%)	4 (57.1%)	0 (0%)	7 (100%)
						
*Tumour resection (%)*						
Radical (R0)	60	7 (11.7%)	8 (13.3%)	45 (75.0%)	9 (15.0%)	51 (85.0%)
Not radical (R1)	11	1 (9.1%)	2 (18.2%)	8 (72.7%)	1 (9.1%)	10 (90.1%)
Not radical (R2)	7	2 (28.6%)	1 (14.3%)	4 (57.1%)	0 (0%)	7 (100%)
						
*Tumour grading (%)*						
G1	7	1 (14.3%)	0 (0%)	6 (85.7%)	2 (28.6%)	5 (71.4%)
G2	30	5 (16.7%)	4 (13.3%)	21 (70%)	4 (13.3%)	26 (86.7%)
G3	41	4 (9.7%)	7 (17.1%)	30 (73,2%)	4 (9.7%)	37 (90.3%)
						
*Patients at follow-up (%)*						
Alive^b^	10	0 (0%)	2 (20.0%)	8 (80.0%)	10	
Dead^c^	68	10 (14.7%)	9 (13.2%)	49 (72.1%)		68

Data are the number of patients.

a Staining patterns: CP–isolated cytoplasmatic staining; CP/nucleus–simultaneous cytoplasmatic and nuclear staining.

b After an average observation time of 15.99 (range 1–61) months.

c Patients died after an average of 15.26 (range 1–49) months, four patients died from non-tumour-related causes.
