# Supplementary figures and images for: The stem cell-associated Hiwi gene in human adenocarcinoma of the pancreas: expression and risk of tumour-related death
Source: Br J Cancer. 2008 Sep 9;99(7):1083–8. doi: 10.1038/sj.bjc.6604653 (PMC2567072; doi:10.1038/sj.bjc.6604653)

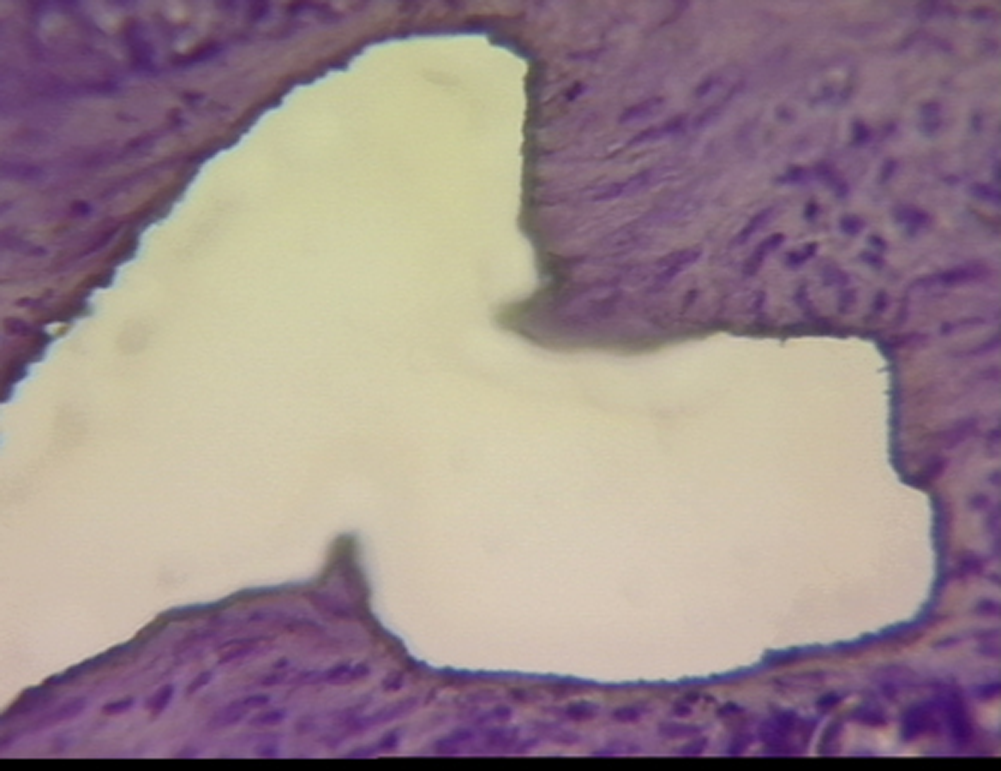

Supplement: Supplementary Figure 1 [file 6604653x1.tif]

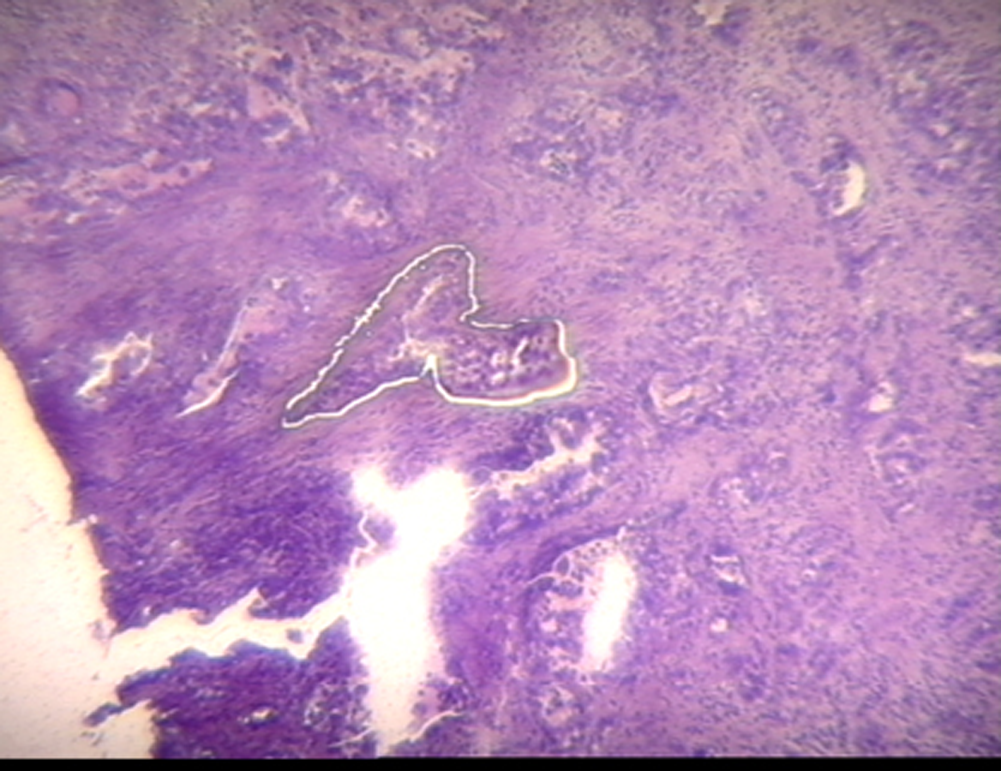

Supplement: Supplementary Figure 2 [file 6604653x2.tif]

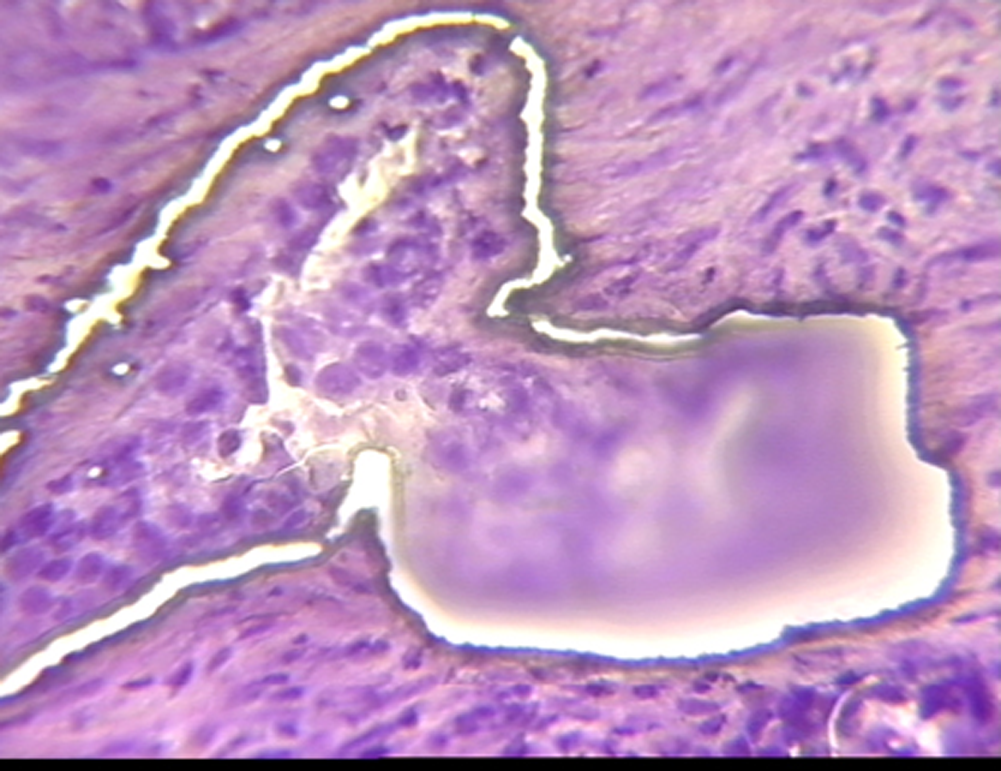

Supplement: Supplementary Figure 3 [file 6604653x3.tif]

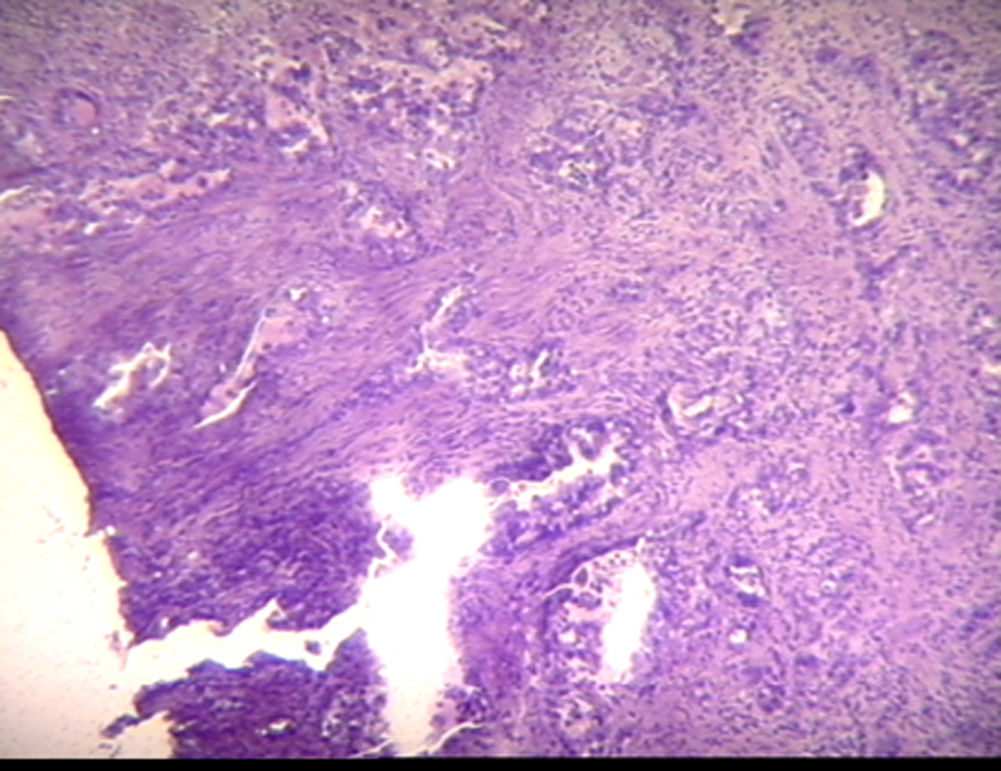

Supplement: Supplementary Figure 4 [file 6604653x4.tif]

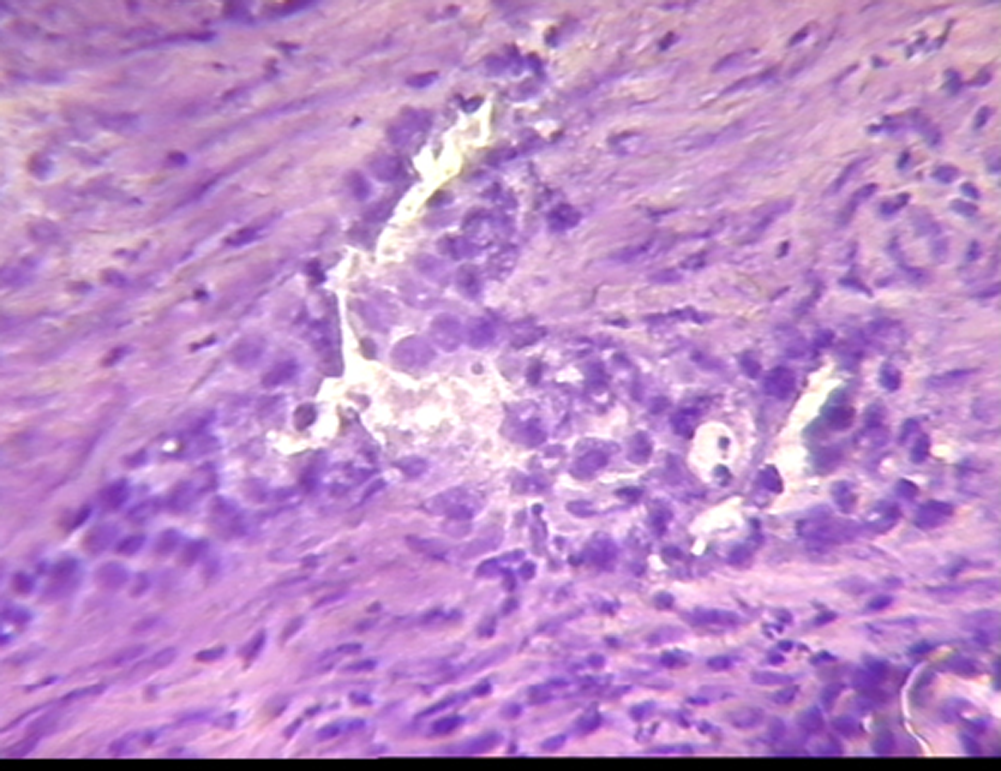

Supplement: Supplementary Figure 5 [file 6604653x5.tif]

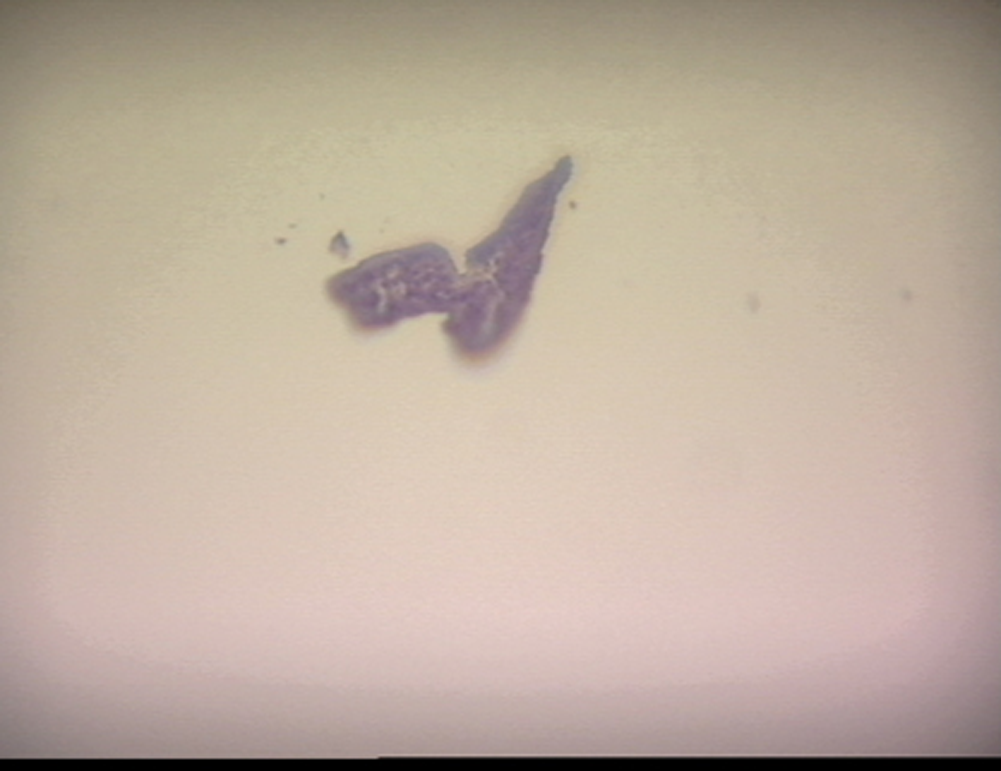

Supplement: Supplementary Figure 6 [file 6604653x6.tif]

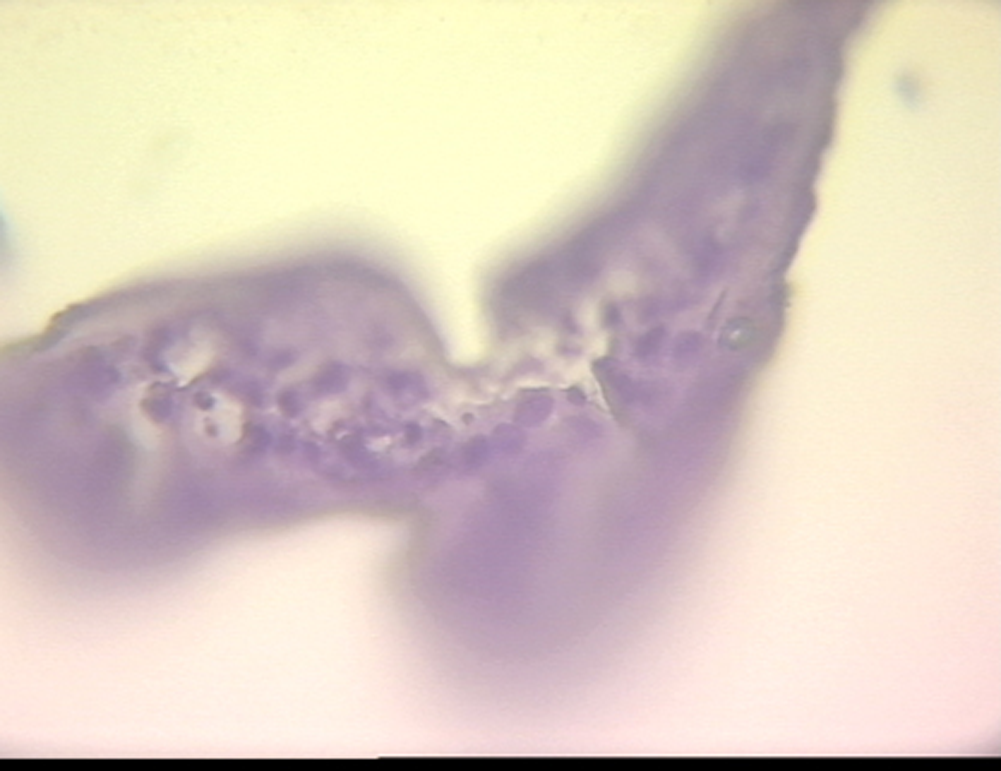

Supplement: Supplementary Figure 7 [file 6604653x7.tif]
