# Supplementary material for: The stem cell-associated Hiwi gene in human adenocarcinoma of the pancreas: expression and risk of tumour-related death
Source: Br J Cancer. 2008 Sep 9;99(7):1083–8. doi: 10.1038/sj.bjc.6604653 (PMC2567072; doi:10.1038/sj.bjc.6604653)
Supplement: Supplementary Information [file 6604653x8.doc]

**Supplementary Figures** Microdissection performed on cresyl violet-stained PDAC tissues.
